# (*E*)-4-Bromo-*N*′-(4-hy­droxy-3-meth­oxy­benzyl­idene)benzohydrazide monohydrate

**DOI:** 10.1107/S160053681201032X

**Published:** 2012-03-14

**Authors:** Jirapa Horkaew, Suchada Chantrapromma, Teerasak Anantapong, Akkharawit Kanjana-Opas, Hoong-Kun Fun

**Affiliations:** a Department of Chemistry and Center of Excellence for Innovation in Chemistry, Faculty of Science, Prince of Songkla University, Hat-Yai, Songkhla 90112, Thailand; b Crystal Materials Research Unit, Department of Chemistry, Faculty of Science, Prince of Songkla University, Hat-Yai, Songkhla 90112, Thailand; c Department of Biotechnology, Faculty of Agro-Industry, Prince of Songkla University, Hat-Yai, Songkhla 90112, Thailand; d X-ray Crystallography Unit, School of Physics, Universiti Sains Malaysia, 11800 USM, Penang, Malaysia

## Abstract

In the title compound, C_15_H_13_BrN_2_O_3_·H_2_O, the dihedral angle between the two benzene rings is 13.92 (6)°. The meth­oxy group of the 4-hy­droxy-3-meth­oxy­phenyl is almost coplanar with its bound benzene ring, as seen by the C_meth­yl_—O—C—C torsion angle of −0.35 (16)°. In the crystal, mol­ecules are linked into a three-dimensional network by N—H⋯O, O—H⋯N and O—H⋯O hydrogen bonds and also weak C—H⋯O inter­actions. A short C⋯O contact of 3.0191 (15) Å is also present.

## Related literature

For bond-length data, see: Allen *et al.* (1987 ▶). For related structures, see: Fun *et al.* (2011 ▶); Horkaew *et al.* (2011 ▶); Promdet *et al.* (2011 ▶). For background and applications of benzohydrazide derivatives, see: Loncle *et al.* (2004 ▶); Raj *et al.* (2007 ▶). For the stability of the temperature controller used in the data collection, see Cosier & Glazer (1986 ▶). 
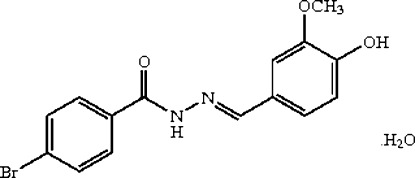


## Experimental

### 

#### Crystal data

C_15_H_13_BrN_2_O_3_·H_2_O
*M* *_r_* = 367.19
Monoclinic, 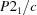

*a* = 7.9772 (7) Å
*b* = 21.446 (2) Å
*c* = 10.3928 (7) Å
β = 119.479 (5)°
*V* = 1547.8 (2) Å^3^
*Z* = 4
Mo *K*α radiation
μ = 2.68 mm^−1^
*T* = 100 K
0.58 × 0.21 × 0.11 mm

#### Data collection

Bruker APEX DUO CCD area-detector diffractometer
Absorption correction: multi-scan (*SADABS*; Bruker, 2009 ▶) *T*_min_ = 0.306, *T*_max_ = 0.756
18815 measured reflections
5602 independent reflections
4894 reflections with *I* > 2σ(*I*)
*R*_int_ = 0.024

#### Refinement

*R*[*F* ^2^ > 2σ(*F* ^2^)] = 0.025
*wR*(*F* ^2^) = 0.065
*S* = 1.04
5602 reflections
200 parameters
H-atom parameters constrained
Δρ_max_ = 0.54 e Å^−3^
Δρ_min_ = −0.54 e Å^−3^

### 

Data collection: *APEX2* (Bruker, 2009 ▶); cell refinement: *SAINT* (Bruker, 2009 ▶); data reduction: *SAINT*; program(s) used to solve structure: *SHELXTL* (Sheldrick, 2008 ▶); program(s) used to refine structure: *SHELXTL*; molecular graphics: *SHELXTL*; software used to prepare material for publication: *SHELXTL* and *PLATON* (Spek, 2009) ▶.

## Supplementary Material

Crystal structure: contains datablock(s) global, I. DOI: 10.1107/S160053681201032X/fj2521sup1.cif

Structure factors: contains datablock(s) I. DOI: 10.1107/S160053681201032X/fj2521Isup2.hkl

Supplementary material file. DOI: 10.1107/S160053681201032X/fj2521Isup3.cml

Additional supplementary materials:  crystallographic information; 3D view; checkCIF report

## Figures and Tables

**Table 1:** Hydrogen-bond geometry (Å, °)

*D*—H⋯*A*	*D*—H	H⋯*A*	*D*⋯*A*	*D*—H⋯*A*
O3—H1*O*3⋯O1*W*^i^	0.80	1.79	2.5867 (14)	170
N1—H1*N*1⋯O3^ii^	0.86	2.18	3.0107 (16)	162
O1*W*—H1*OW*⋯O1^iii^	0.82	1.93	2.7409 (14)	171
O1*W*—H2*OW*⋯O1^iv^	0.78	2.16	2.8883 (14)	154
O1*W*—H2*OW*⋯N2^iv^	0.78	2.49	3.0971 (16)	136
C6—H6*A*⋯O3^ii^	0.95	2.59	3.4832 (15)	156
C8—H8*A*⋯O3^ii^	0.95	2.40	3.2604 (17)	150
C10—H10*A*⋯O1*W*^v^	0.95	2.45	3.3933 (15)	172

Symmetry codes: (i) 
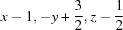
; (ii) 
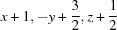
; (iii) 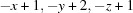; (iv) 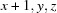; (v) 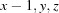.

**Fig. 1.**
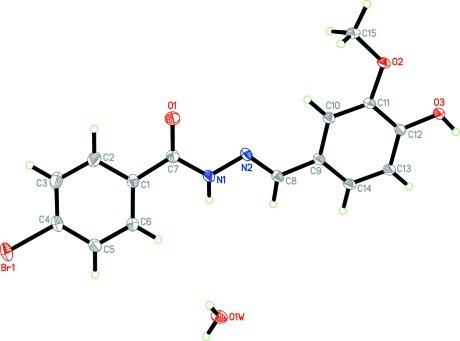
The molecular structure of the title compound, showing 55% probability displacement ellipsoids and the atom-numbering scheme.

**Fig. 2.**
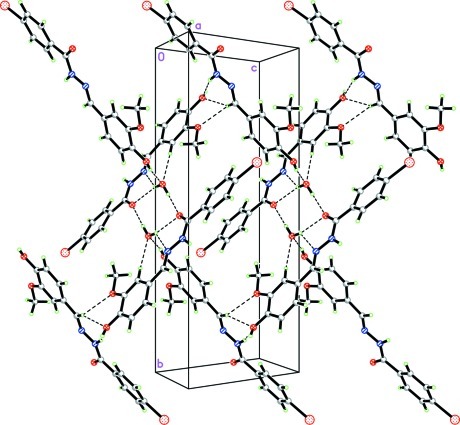
The crystal packing of the title compound viewed approximately along the a axis, showing 3D network. Hydrogen bonds were drawn as dashed lines.

## supplementary crystallographic information

### Comment

As part of our study on bioactivity of hydrazone and benzohydrazide derivatives, the title compound is one of the several benzohydrazide derivatives which were synthesized and tested for biological activity. It have been known that some benzohydrazides possess various biological properties, such as antibacterial and antifungal (Loncle *et al.*, 2004), and antiproliferative (Raj *et al.*, 2007) activities. We have previously reported some crystal structures of this category of compounds (Fun *et al.*, 2011; Horkaew *et al.*, 2011; Promdet *et al.*, 2011). The title compound (I) was synthesized in order to study the effect of functional groups and their positions on their bioactivities by comparing with the closely related structures in our research project. (I) was screened for antibacterial and antioxidant activities. Our biological testing found that (I) exhibits potent antioxidant activity whereas inactive against the tested bacteria strains which are *Bacillus subtilis*, *Enterococcus faecalis*, *Staphylococcus aureus*, Methicillin-Resistant *Staphylococcus aureus*, Vancomycin-Resistant *Enterococcus faecalis*, *Pseudomonas aeruginosa*, *Salmonella typhi* and *Shigella sonnei*. Herein we report the crystal structure of (I).

The molecule of the title benzohydrazide derivative (Fig. 1), C_15_H_13_BrN_2_O_3_.H_2_O, comprises of a molecule of benzohydrazide and one water solvent molecule. The molecule of benzohydrazide exists in a *trans*-configuration with respect to the C8═N2 bond [1.2853 (14) Å] and the torsion angle N1–N2–C8–C9 = 178.54 (10)°. The molecule is twisted with the dihedral angle between the two phenyl rings being 13.92 (6)°. The methoxy group of the 4-hydroxy-3-methoxyphenyl is co-planar with its bound benzene ring [C15–O2–C11–C10 = 0.35 (16)°].

The middle bridge fragment (O1/C7/N1/N2/C8) is essentially planar with the torsion angle N2–N1–C7–O1 = -0.21 (17)°. The mean plane through this bridge makes the dihedral angles of 12.71 (7) and 1.25 (7)° with the 4-bromophenyl and 4 benzene rings, respectively. The methoxy group of 4-hydroxy-3-methoxyphenyl is co-planar with its bound benzene ring with the torsion angle C15–O2–C11–C10 = 0.35 (16)° and the r.m.s 0.0063 (2) Å for the eight non H atoms. Bond distances are in normal ranges (Allen *et al.*, 1987) and are comparable with the related structures (Fun *et al.*, 2011; Horkaew *et al.*, 2011; Promdet *et al.*, 2011).

In the crystal packing (Fig. 2), the molecules are linked by N—H···O, O—H···N and O—H···O hydrogen bonds together with weak C—H···O interactions (Table 1) into a three dimensional network. A C8···O2^i^[3.0191 (15) Å] short contact was presented.

### Experimental

The title compound (I) was prepared by dissolving 4-bromobenzohydrazide (2 mmol, 0.43 g) in ethanol (15 ml). The solution of 4-hydroxy-3-methoxy-benzaldehyde (2 mmol, 0.30 g) in ethanol (15 ml) was then added slowly to the reaction. The mixture was refluxed for around 5 hr and the white solid of the product that appeared was collected by filtration, washed with ethanol and dried in air. Colorless block-shaped single crystals of the title compound suitable for *X*-ray structure determination were recrystallized from methanol by slow evaporation of the solvent at room temperature after several days, Mp. 513 K (decomposed).

### Refinement

All H atoms were positioned geometrically and allowed to ride on their parent atoms, with d(N-H) = 0. 86 Å, d(O-H) = 0.80 Å for hydroxy and 0.78 and 0.82 Å for water, d(C-H) = 0.95 Å for aromatic and CH and 0.98 Å for CH_3_ atoms. The *U*_iso_ values were constrained to be 1.5*U*_eq_ of the carrier atom for methyl H atoms and 1.2*U*_eq_ for the remaining H atoms. A rotating group model was used for the methyl groups.

### Crystal data

**Table:**

C_15_H_13_BrN_2_O_3_·H_2_O	*F*(000) = 744
*M**_r_* = 367.19	*D*_x_ = 1.576 Mg m^−^^3^
Monoclinic, *P*2_1_/*c*	Melting point > 513 K
Hall symbol: -P 2ybc	Mo *K*α radiation, λ = 0.71073 Å
*a* = 7.9772 (7) Å	Cell parameters from 5602 reflections
*b* = 21.446 (2) Å	θ = 2.4–32.6°
*c* = 10.3928 (7) Å	µ = 2.68 mm^−^^1^
β = 119.479 (5)°	*T* = 100 K
*V* = 1547.8 (2) Å^3^	Block, colorless
*Z* = 4	0.58 × 0.21 × 0.11 mm

### Data collection

**Table:**

Bruker APEX DUO CCD area-detector diffractometer	5602 independent reflections
Radiation source: sealed tube	4894 reflections with *I* > 2σ(*I*)
Graphite monochromator	*R*_int_ = 0.024
φ and ω scans	θ_max_ = 32.6°, θ_min_ = 2.4°
Absorption correction: multi-scan (*SADABS*; Bruker, 2009)	*h* = −11→12
*T*_min_ = 0.306, *T*_max_ = 0.756	*k* = −29→32
18815 measured reflections	*l* = −15→15

### Refinement

**Table:**

Refinement on *F*^2^	Primary atom site location: structure-invariant direct methods
Least-squares matrix: full	Secondary atom site location: difference Fourier map
*R*[*F*^2^ > 2σ(*F*^2^)] = 0.025	Hydrogen site location: inferred from neighbouring sites
*wR*(*F*^2^) = 0.065	H-atom parameters constrained
*S* = 1.04	*w* = 1/[σ^2^(*F*_o_^2^) + (0.0304*P*)^2^ + 0.5617*P*] where *P* = (*F*_o_^2^ + 2*F*_c_^2^)/3
5602 reflections	(Δ/σ)_max_ = 0.009
200 parameters	Δρ_max_ = 0.54 e Å^−^^3^
0 restraints	Δρ_min_ = −0.54 e Å^−^^3^

### Special details

**Table:**

Experimental. The crystal was placed in the cold stream of an Oxford Cryosystems Cobra open-flow nitrogen cryostat (Cosier & Glazer, 1986) operating at 100.0 (1) K.
Geometry. All esds (except the esd in the dihedral angle between two l.s. planes) are estimated using the full covariance matrix. The cell esds are taken into account individually in the estimation of esds in distances, angles and torsion angles; correlations between esds in cell parameters are only used when they are defined by crystal symmetry. An approximate (isotropic) treatment of cell esds is used for estimating esds involving l.s. planes.
Refinement. Refinement of F^2^ against ALL reflections. The weighted R-factor wR and goodness of fit S are based on F^2^, conventional R-factors R are based on F, with F set to zero for negative F^2^. The threshold expression of F^2^ > 2sigma(F^2^) is used only for calculating R-factors(gt) etc. and is not relevant to the choice of reflections for refinement. R-factors based on F^2^ are statistically about twice as large as those based on F, and R- factors based on ALL data will be even larger.

### Fractional atomic coordinates and isotropic or equivalent isotropic displacement parameters (Å^2^)

**Table:**

	*x*	*y*	*z*	*U*_iso_*/*U*_eq_	
Br1	1.165101 (18)	1.138681 (6)	1.032760 (13)	0.02180 (4)	
O1	0.31020 (12)	0.98324 (4)	0.62686 (11)	0.02206 (18)	
O2	−0.26566 (11)	0.74388 (4)	0.19684 (10)	0.01696 (15)	
O3	−0.14414 (12)	0.64604 (4)	0.11703 (10)	0.01614 (15)	
H1O3	−0.1053	0.6238	0.0752	0.024*	
N1	0.50431 (13)	0.91557 (4)	0.59638 (11)	0.01464 (16)	
H1N1	0.6171	0.9016	0.6218	0.018*	
N2	0.34933 (13)	0.87658 (4)	0.51278 (11)	0.01445 (16)	
C1	0.64474 (16)	1.00888 (5)	0.74049 (12)	0.01489 (18)	
C2	0.62537 (18)	1.05722 (6)	0.82220 (15)	0.0223 (2)	
H2A	0.5052	1.0635	0.8185	0.027*	
C3	0.77907 (19)	1.09623 (6)	0.90869 (15)	0.0233 (2)	
H3A	0.7652	1.1289	0.9644	0.028*	
C4	0.95314 (17)	1.08667 (5)	0.91237 (13)	0.0176 (2)	
C5	0.97598 (17)	1.03960 (6)	0.83136 (13)	0.0186 (2)	
H5A	1.0960	1.0339	0.8344	0.022*	
C6	0.82108 (17)	1.00077 (5)	0.74535 (13)	0.0178 (2)	
H6A	0.8356	0.9684	0.6893	0.021*	
C7	0.47302 (16)	0.96867 (5)	0.65048 (12)	0.01518 (19)	
C8	0.39443 (15)	0.82791 (5)	0.46425 (12)	0.01448 (18)	
H8A	0.5240	0.8228	0.4859	0.017*	
C9	0.25378 (15)	0.78044 (5)	0.37726 (12)	0.01375 (18)	
C10	0.05771 (15)	0.78701 (5)	0.33432 (12)	0.01418 (18)	
H10A	0.0146	0.8225	0.3646	0.017*	
C11	−0.07205 (15)	0.74162 (5)	0.24783 (12)	0.01331 (18)	
C12	−0.00870 (15)	0.68869 (5)	0.20363 (12)	0.01369 (18)	
C13	0.18522 (16)	0.68186 (5)	0.24792 (13)	0.01568 (19)	
H13A	0.2288	0.6460	0.2194	0.019*	
C14	0.31573 (16)	0.72779 (5)	0.33436 (13)	0.01619 (19)	
H14A	0.4484	0.7231	0.3643	0.019*	
C15	−0.33607 (17)	0.79716 (6)	0.23765 (15)	0.0201 (2)	
H15A	−0.4763	0.7943	0.1930	0.030*	
H15B	−0.3026	0.8350	0.2024	0.030*	
H15C	−0.2776	0.7988	0.3455	0.030*	
O1W	0.94692 (12)	0.92018 (4)	0.45250 (10)	0.01886 (16)	
H1OW	0.8746	0.9503	0.4230	0.028*	
H2OW	1.0559	0.9287	0.4881	0.028*	

### Atomic displacement parameters (Å^2^)

**Table:**

	*U*^11^	*U*^22^	*U*^33^	*U*^12^	*U*^13^	*U*^23^
Br1	0.02481 (7)	0.01815 (6)	0.01611 (6)	−0.00733 (4)	0.00520 (5)	−0.00200 (4)
O1	0.0137 (4)	0.0200 (4)	0.0292 (5)	0.0032 (3)	0.0080 (3)	−0.0033 (3)
O2	0.0091 (3)	0.0177 (4)	0.0223 (4)	−0.0001 (3)	0.0064 (3)	−0.0044 (3)
O3	0.0129 (3)	0.0161 (4)	0.0196 (4)	−0.0031 (3)	0.0081 (3)	−0.0051 (3)
N1	0.0099 (4)	0.0142 (4)	0.0170 (4)	−0.0005 (3)	0.0045 (3)	−0.0019 (3)
N2	0.0109 (4)	0.0147 (4)	0.0146 (4)	−0.0018 (3)	0.0039 (3)	−0.0008 (3)
C1	0.0152 (5)	0.0122 (4)	0.0150 (4)	0.0006 (3)	0.0056 (4)	0.0007 (3)
C2	0.0182 (5)	0.0206 (5)	0.0256 (6)	0.0023 (4)	0.0088 (5)	−0.0057 (4)
C3	0.0222 (6)	0.0194 (5)	0.0238 (6)	0.0011 (4)	0.0080 (5)	−0.0064 (4)
C4	0.0199 (5)	0.0145 (5)	0.0138 (5)	−0.0031 (4)	0.0046 (4)	−0.0002 (4)
C5	0.0184 (5)	0.0189 (5)	0.0194 (5)	−0.0048 (4)	0.0100 (4)	−0.0032 (4)
C6	0.0186 (5)	0.0166 (5)	0.0192 (5)	−0.0034 (4)	0.0101 (4)	−0.0039 (4)
C7	0.0137 (4)	0.0141 (4)	0.0154 (5)	0.0013 (3)	0.0053 (4)	0.0007 (4)
C8	0.0106 (4)	0.0154 (4)	0.0152 (5)	0.0000 (3)	0.0046 (4)	0.0007 (4)
C9	0.0112 (4)	0.0141 (4)	0.0145 (4)	−0.0005 (3)	0.0052 (4)	−0.0001 (3)
C10	0.0121 (4)	0.0143 (4)	0.0150 (5)	0.0001 (3)	0.0058 (4)	−0.0007 (4)
C11	0.0100 (4)	0.0147 (4)	0.0145 (4)	0.0003 (3)	0.0054 (4)	0.0006 (3)
C12	0.0116 (4)	0.0141 (4)	0.0142 (4)	−0.0015 (3)	0.0055 (4)	−0.0009 (3)
C13	0.0131 (4)	0.0148 (4)	0.0192 (5)	0.0006 (4)	0.0080 (4)	−0.0020 (4)
C14	0.0109 (4)	0.0172 (5)	0.0198 (5)	−0.0002 (4)	0.0070 (4)	−0.0013 (4)
C15	0.0134 (5)	0.0214 (5)	0.0261 (6)	0.0014 (4)	0.0103 (4)	−0.0048 (4)
O1W	0.0147 (4)	0.0161 (4)	0.0266 (4)	0.0023 (3)	0.0108 (3)	0.0042 (3)

### Geometric parameters (Å, º)

**Table:**

Br1—C4	1.8939 (12)	C5—H5A	0.9500
O1—C7	1.2374 (14)	C6—H6A	0.9500
O2—C11	1.3650 (13)	C8—C9	1.4541 (15)
O2—C15	1.4266 (14)	C8—H8A	0.9500
O3—C12	1.3627 (13)	C9—C14	1.3911 (15)
O3—H1O3	0.8032	C9—C10	1.4066 (15)
N1—C7	1.3467 (14)	C10—C11	1.3825 (15)
N1—N2	1.3876 (13)	C10—H10A	0.9500
N1—H1N1	0.8572	C11—C12	1.4082 (15)
N2—C8	1.2853 (14)	C12—C13	1.3876 (15)
C1—C6	1.3935 (16)	C13—C14	1.3930 (16)
C1—C2	1.3959 (16)	C13—H13A	0.9500
C1—C7	1.4947 (16)	C14—H14A	0.9500
C2—C3	1.3879 (18)	C15—H15A	0.9800
C2—H2A	0.9500	C15—H15B	0.9800
C3—C4	1.3854 (18)	C15—H15C	0.9800
C3—H3A	0.9500	O1W—H1OW	0.8179
C4—C5	1.3828 (16)	O1W—H2OW	0.7806
C5—C6	1.3900 (16)		
			
C11—O2—C15	116.68 (9)	N2—C8—H8A	118.9
C12—O3—H1O3	111.4	C9—C8—H8A	118.9
C7—N1—N2	118.68 (9)	C14—C9—C10	119.65 (10)
C7—N1—H1N1	123.2	C14—C9—C8	118.72 (9)
N2—N1—H1N1	117.4	C10—C9—C8	121.63 (10)
C8—N2—N1	113.57 (9)	C11—C10—C9	119.71 (10)
C6—C1—C2	118.85 (11)	C11—C10—H10A	120.1
C6—C1—C7	123.27 (10)	C9—C10—H10A	120.1
C2—C1—C7	117.87 (10)	O2—C11—C10	124.71 (10)
C3—C2—C1	120.97 (11)	O2—C11—C12	114.91 (9)
C3—C2—H2A	119.5	C10—C11—C12	120.37 (9)
C1—C2—H2A	119.5	O3—C12—C13	122.71 (10)
C4—C3—C2	118.85 (11)	O3—C12—C11	117.47 (9)
C4—C3—H3A	120.6	C13—C12—C11	119.82 (10)
C2—C3—H3A	120.6	C12—C13—C14	119.78 (10)
C5—C4—C3	121.46 (11)	C12—C13—H13A	120.1
C5—C4—Br1	119.34 (9)	C14—C13—H13A	120.1
C3—C4—Br1	119.20 (9)	C9—C14—C13	120.66 (10)
C4—C5—C6	119.15 (11)	C9—C14—H14A	119.7
C4—C5—H5A	120.4	C13—C14—H14A	119.7
C6—C5—H5A	120.4	O2—C15—H15A	109.5
C5—C6—C1	120.70 (11)	O2—C15—H15B	109.5
C5—C6—H6A	119.6	H15A—C15—H15B	109.5
C1—C6—H6A	119.6	O2—C15—H15C	109.5
O1—C7—N1	121.53 (10)	H15A—C15—H15C	109.5
O1—C7—C1	121.85 (10)	H15B—C15—H15C	109.5
N1—C7—C1	116.62 (9)	H1OW—O1W—H2OW	114.1
N2—C8—C9	122.22 (10)		
			
C7—N1—N2—C8	178.70 (10)	N2—C8—C9—C14	−177.20 (11)
C6—C1—C2—C3	−0.88 (19)	N2—C8—C9—C10	3.67 (17)
C7—C1—C2—C3	179.75 (12)	C14—C9—C10—C11	−1.09 (16)
C1—C2—C3—C4	0.3 (2)	C8—C9—C10—C11	178.04 (10)
C2—C3—C4—C5	0.4 (2)	C15—O2—C11—C10	−0.35 (16)
C2—C3—C4—Br1	−178.89 (10)	C15—O2—C11—C12	−179.25 (10)
C3—C4—C5—C6	−0.52 (19)	C9—C10—C11—O2	−178.33 (10)
Br1—C4—C5—C6	178.78 (9)	C9—C10—C11—C12	0.52 (16)
C4—C5—C6—C1	−0.09 (18)	O2—C11—C12—O3	−0.29 (14)
C2—C1—C6—C5	0.77 (18)	C10—C11—C12—O3	−179.25 (10)
C7—C1—C6—C5	−179.89 (11)	O2—C11—C12—C13	179.38 (10)
N2—N1—C7—O1	−0.21 (17)	C10—C11—C12—C13	0.42 (16)
N2—N1—C7—C1	179.66 (9)	O3—C12—C13—C14	178.87 (10)
C6—C1—C7—O1	−166.86 (12)	C11—C12—C13—C14	−0.78 (17)
C2—C1—C7—O1	12.48 (17)	C10—C9—C14—C13	0.74 (17)
C6—C1—C7—N1	13.26 (16)	C8—C9—C14—C13	−178.42 (11)
C2—C1—C7—N1	−167.40 (11)	C12—C13—C14—C9	0.20 (18)
N1—N2—C8—C9	178.54 (10)		

### Hydrogen-bond geometry (Å, º)

**Table:**

*D*—H···*A*	*D*—H	H···*A*	*D*···*A*	*D*—H···*A*
O3—H1*O*3···O1*W*^i^	0.80	1.79	2.5867 (14)	170
N1—H1*N*1···O3^ii^	0.86	2.18	3.0107 (16)	162
O1*W*—H1*OW*···O1^iii^	0.82	1.93	2.7409 (14)	171
O1*W*—H2*OW*···O1^iv^	0.78	2.16	2.8883 (14)	154
O1*W*—H2*OW*···N2^iv^	0.78	2.49	3.0971 (16)	136
C6—H6*A*···O3^ii^	0.95	2.59	3.4832 (15)	156
C8—H8*A*···O2^ii^	0.95	2.46	3.0191 (15)	118
C8—H8*A*···O3^ii^	0.95	2.40	3.2604 (17)	150
C10—H10*A*···O1*W*^v^	0.95	2.45	3.3933 (15)	172

Symmetry codes: (i) *x*−1, −*y*+3/2, *z*−1/2; (ii) *x*+1, −*y*+3/2, *z*+1/2; (iii) −*x*+1, −*y*+2, −*z*+1; (iv) *x*+1, *y*, *z*; (v) *x*−1, *y*, *z*.
